# Percutaneous implantation of peripherally inserted totally implantable venous access systems in the forearm in adolescent patients

**DOI:** 10.1007/s00247-022-05321-x

**Published:** 2022-04-04

**Authors:** Anne Marie Augustin, Olivia Kertels, Verena Wiegering, Annette Thurner, Ralph Kickuth

**Affiliations:** 1 Department of Diagnostic and Interventional Radiology, University Hospital of Würzburg, Oberdürrbacher Strasse 6, DE 97080 Würzburg, Germany; 2 Department of Pediatrics, University Hospital of Würzburg, Würzburg, Germany

**Keywords:** Adolescents, Central venous catheter, Children, Forearm, Interventional radiology, Totally implantable venous access port, Vascular access

## Abstract

**Background:**

Children with different underlying malignant diseases require long-term central venous access. As for port systems in a pectoral position, peripherally implanted port systems in the forearm revealed high levels of technical and clinical success in adult cohorts.

**Objective:**

To investigate the technical and clinical outcomes of percutaneous central venous port implantation in the forearm in adolescents.

**Materials and methods:**

Between April 2010 and August 2020, 32 children ages 9 to 17 years with underlying malignancy received 35 totally implantable venous access ports (TIVAPs) in the forearm. All venous port systems were peripherally inserted under ultrasound guidance. Correct catheter placement was controlled by fluoroscopy. As primary endpoints, the technical success, rate of complications and catheter maintenance were analyzed. Secondary endpoints were the side of implantation, vein of catheter access, laboratory results on the day of the procedure, procedural radiation exposure, amount of contrast agent and reasons for port device removal.

**Results:**

Percutaneous TIVAP placement under sonographic guidance was technically successful in 34 of 35 procedures (97.1%). Procedure-related complications did not occur. During the follow-up, 13,684 catheter days were analyzed, revealing 11 complications (0.8 per 1,000 catheter-duration days), Of these 11 complications, 7 were major and 10 occurred late. In seven cases, the port device had to be removed; removal-related complications did not occur.

**Conclusion:**

Peripheral TIVAP placement in the forearms of children is a feasible, effective and safe technique with good midterm outcome. As results are comparable with standard access routes, this technique may be offered as an alternative when intermittent venous access is required.

## Introduction

Safe, reliable and long-term central venous access is of utmost importance in the treatment of patients with underlying diseases [1]. Totally implantable venous access ports (TIVAP) are long-term central venous catheter systems that carry the advantage of avoiding repeated venous punctures while interfering only mildly with patients´ daily life activities [2]. Generally, TIVAP systems should be considered when repetitive administration of chemotherapeutics, antibiotics, blood products or parenteral nutrition is needed [3, 4]. Chemotherapeutic agents in particular frequently irritate the veins, making it even more difficult to establish a safe venous access. In this context, long-term central venous access devices significantly facilitate the intermittent administration of chemotherapeutic agents. Being an alternative for TIVAPs, externalized and tunneled catheters like Hickman catheters carry an increased risk for catheter-associated infections, dislocations and leakages [5]. Furthermore, such devices restrict activities of daily life, e.g., bathing or swimming. Consequently, TIVAPs have widely replaced externally tunneled systems.

Previous studies of central venous port systems in the forearm in predominantly adult cohorts reported high levels of feasibility and low early and late complication rates [6, 7]. However, limited data exist regarding the application of peripherally inserted port systems in children [8]. Due to age-related vulnerabilities and needs distinct from those of adults, children represent a specific patient cohort. Among others, these differences include physiological development, dependency on adults and the nature of underlying diseases. As a result, epidemiology and the type of adverse events differ between children and adults [9, 10]. Therefore, the aim of the present study was to evaluate the technical and clinical outcomes of peripherally inserted TIVAPs in the forearm in adolescents. To the best of our knowledge, the implantation of such systems in the forearm of pediatric patients has not yet been explicitly described.

## Materials and methods

### Study collective

A retrospective review of our interventional radiology department archives between April 2010 and August 2020 yielded 32 consecutive pediatric patients (11 male) who had undergone ultrasound-guided peripheral port system implantation in the forearm. Three patients received two ports, resulting in a total of 35 procedures.

The median age of the patients was 15 years (range: 9–17 years). All included patients were referred for peripheral port implantation because of an underlying malignant disease for intravenous administration of chemotherapy. The oncological diseases included hematopoietic malignancies in 19 and solid tumors in 13 patients. Patient demographic characteristics are in Table 1. All included patients were treated as part of routine care and their parents or legal guardians gave written informed consent for the procedure. The local institutional review board waived its approval before conducting this retrospective study.

**Table 1:** Patient demographic data (*n*=32)

Age (years)	
Mean (±SD)	14.8 (±2.3)
Range	9–17
Gender	*n* (%)
Male	11 (34.4)
Female	21 (64.6)
Underlying malignancy	*n* (%)
Classical Hodgkin lymphoma	14 (43.8)
T cell lymphoma	4 (12.5)
Acute lymphoblastic leukemia	2 (6.3)
Astrocytoma	2 (6.3)
Choroid plexus carcinoma	1 (3.1)
Epitheloid sarcoma	1 (3.1)
Extraosseous Ewing sarcoma	1 (3.1)
Ganglioglioma	1 (3.1)
Glioblastoma	1 (3.1)
Optic nerve glioma	1 (3.1)

*SD* standard deviation

Indications for implantation of TIVAPs in the forearm included the need for long-term venous access for the administration of less aggressive chemotherapeutic agents with special regard to the coagulation cascade. In addition, only children ≥8 years of age were included on their or their parents’ personal request. Girls (mainly but not exclusively) rejected pectoral port systems, to avoid scar formation in the cleavage and/or exposure of their upper bodies when accessing their port systems. Otherwise, pectoral port placement is considered the standard approach.

### Procedure

All procedures were performed in our local angiography suite (Siemens; Axiom Artis Zee, Forchheim, Germany) by interventional radiologists with differing years of experience, including interventional radiologists in training. Whenever possible, the port system was implanted contralateral to the dominant hand. A possible reason for proceeding differently was the presence of large tumor masses that might impede the placement of the catheter centrally from the appropriate side. In this context, preexisting computed tomography (CT) or magnetic resonance imaging (MRI) was evaluated to exclude mediastinal tumor masses and to identify direct or indirect signs of thrombosis in the venous insertion route.

**Fig. 1:**
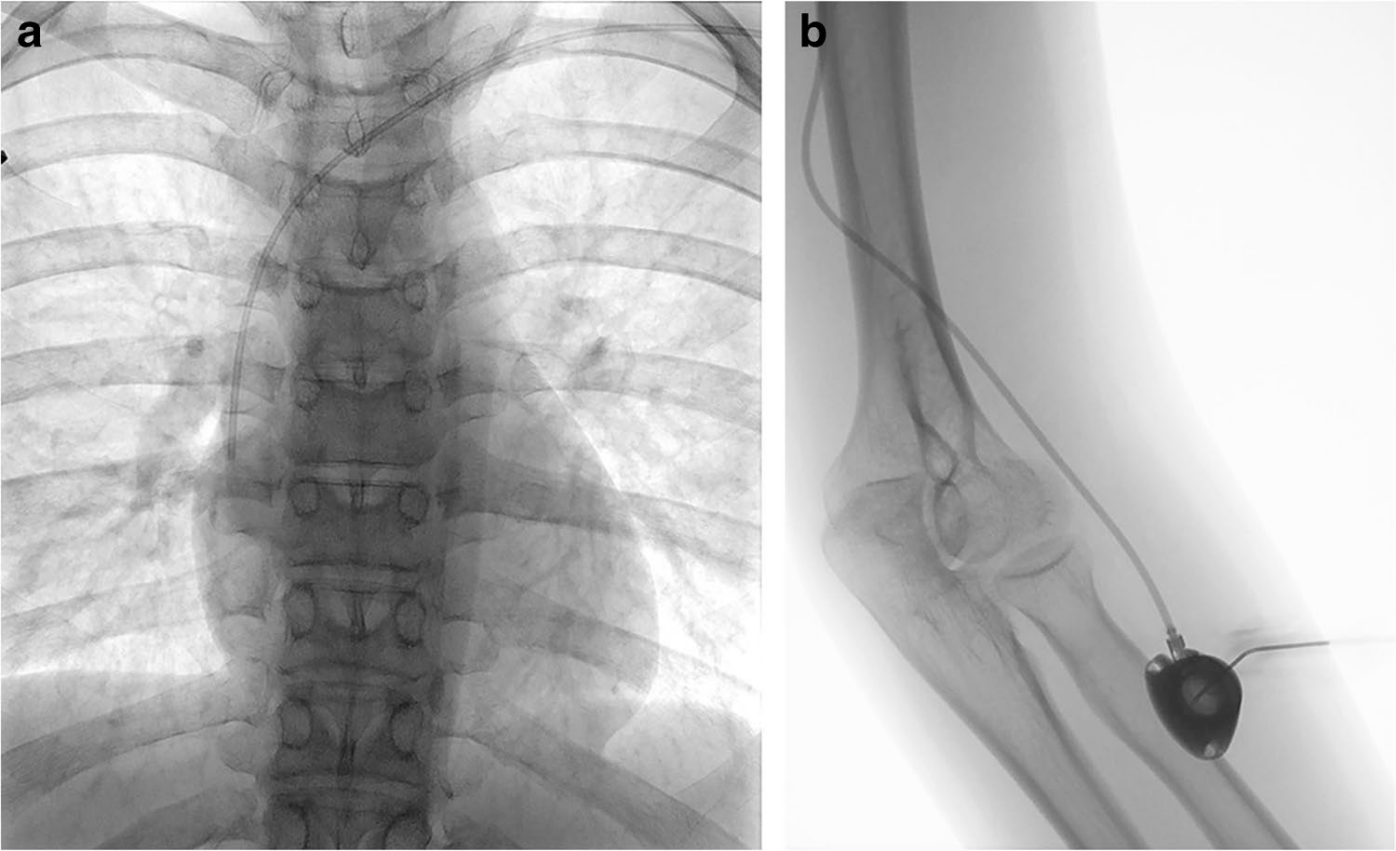
Port implantation in the left forearm in a 16-year-old girl with a rhabdomyosarcoma. **a** Posteroanterior fluoroscopy confirms correct placement of the port catheter at the level of the central superior vena cava, just above the right atrium. **b** Oblique projection fluoroscopy image shows an implanted port chamber, with the port-puncture needle inserted in the proximal lateral forearm distal to the cubital fossa. Injection of a small volume of contrast agent proved the correct placement and connection of the port system

Most procedures (25/35) were performed under local anaesthetic without the need for further sedation. Six patients required sedation with intravenous 2 mg midazolam and four procedures were performed under general anaesthetic. No sedation or anaesthesia-related complications occurred. Different port devices were used, most of them (3/4) approved for high-pressure injections (P.A.S. Port T2 POWER P.A.C., 6F; Smith Medical MD, St. Paul, MN; Celsite Babyport 4.5 F; B. Braun Medical, Boulogne Cedex, France; Celsite, 6.5F; B. Braun Medical, Boulogne Cedex, France; Vital Port Mini System, 5 F; Cook, Bjaeverskov, Denmark). The heights of the port chambers ranged from 7.2 to 13.7 mm. Puncture and implantation sites were sterilely prepared (SkinseptG, Ecolab, Austria), and draped. Additionally, a blood pressure cuff was placed on the upper arm. Under ultrasound control and with the blood pressure cuff inflated below the systolic pressure, the basilic vein (at the distal upper arm) was punctured using an 18 G needle. In this context, a sterile covered high-frequency linear array (8 MHz) probe was utilized (Siemens ACUSON Freestyle). The orientation over the vein was at the discretion of the performing interventional radiologist, although a transverse orientation was used by most. The needle was placed in the middle of the transducer, advanced through the subcutaneous tissue, then the anterior vessel wall punctured under sonographic control. Just before penetrating the anterior vessel wall, it proved beneficial to reduce the needle angle, especially in more superficial veins. With the blood pressure cuff still inflated, either the needle sheath was directly advanced, or alternatively, a 0.014-in. nitinol guidewire (Medtronics, Minneapolis, MN, USA) was inserted prior to guidewire-supported insertion of the sheath. In cases of a noncompressible or small diameter basilic vein (<2 mm), the cephalic or brachial vein was used for venous access. After successful vein puncture, a 0.035-in. guidewire was introduced, with advancement through the vein fluoroscopically guided. The preference for the basilic vein is based on its relatively straightforward accessibility for puncture and subcutaneous tunneling, as well as the low risk of inadvertent arterial puncture or damage to the median nerve. Local anaesthetic was applied to the vascular access site, a small incision was then made followed by wire-supported exchange of the introducer needle for a peel-away sheath. In 31 procedures (88.6%), intravenous antibiotic (a first- or second-generation broad-spectrum cephalosporin) was administered periprocedurally via the sheath for infection prophylaxis. In patients allergic to penicillin or other antibiotics, the choice of an alternative antibiotic was made by the referring physician. Patients already receiving appropriate antibiotic therapy for another purpose received no further prophylaxis. Subsequently, local anaesthetic was applied on the proximal lateral forearm distal to the cubital fossa and in the location of the intended catheter tunnel. A 2- to 3-cm wide incision was made at the anesthetized site and a subcutaneous pocket for the port chamber created by means of a blunt dissection technique. Following this, the catheter was introduced via the peel-away sheath and placed in the superior vena cava under pulsed fluoroscopic guidance (7.5 pulses per s). In cases of difficult placement of the catheter within the superior vena cava, it often proved beneficial to advance the catheter with the patient’s head rotated toward the contralateral side while inhaling deeply. If these strategies still failed to allow catheter placement, a hydrophilic guidewire with a J angled tip was advanced through the catheter lumen into the superior vena cava and the catheter was placed over the wire. After tunneling the distance between the vascular access site and port chamber pocket, the final catheter position was achieved. The catheter was then shortened to an adequate length and connected to the port chamber, which was subsequently implanted subcutaneously. Port chambers were not routinely fixed with sutures in the subcutaneous pocket. The pocket was closed by means of subcutaneous (resorbable 4–0 Vicryl) and cutaneous sutures (3–0 Prolene). Closure of the incision at the vascular access site was performed with a single cutaneous suture. Using the included port-puncture needle, the port system was accessed, and the ability to aspirate blood was confirmed. Finally, the correct catheter position and connection to the port chamber were verified fluoroscopically with the injection of a small volume of contrast agent (Imeron 300; Bracco Imaging, Milano, Italy) (Fig. 1). The port system was then carefully flushed and locked with heparinized sodium chloride. Removal of cutaneous sutures occurred after 10–14 days. Patients and their parents were advised to contact the outpatient clinic immediately at the onset of any signs of infection. According to our in-house guidelines, a heparin lock is performed after each use of a port system. The approach outlined above did not change substantially over the study period.

### Data analysis and endpoint definition

Medical and radiologic records were retrospectively reviewed for information regarding the procedural outcome. Primary endpoints were technical success, clinical outcome and early and late minor and major complications.

Technical success was defined as correct catheter placement in the central venous system, documented by fluoroscopy and satisfactory port system function at the end of the intervention. Clinical outcome was expressed by device failure characterized by any limitation in catheter function despite technically successful catheter placement. The initial device service interval was described as the number of catheter days from the implantation procedure until removal at completion of therapy, patients´ demise, end of study with the catheter still functioning or device failure [6].

Procedure-related complications were evaluated on the basis of the reporting standards of the Society of Interventional Radiology for central venous access [4]. Minor complications included those resulting in (A) no therapy and no consequence or (B) nominal therapy and no consequence including overnight admission for observation only. Major complications included (C) those requiring therapy or minor hospitalization (<48 h), (D) those requiring major therapy, unplanned increase in level of care or prolonged hospitalization (>48 h), (E) those resulting in permanent adverse sequelae and (F) those resulting in treatment-related mortality. Additionally, complications were graded as early occurring within the first 30 days after the procedure and as late when occurring after 30 days of the implantation.

Secondary endpoints were the implantation side, the catheter access vein, laboratory results with values of leukocytes, C-reactive protein (CRP) and partial thromboplastin time (PTT) on the day of the intervention, procedural fluoroscopy time (FT) and dose area product (DAP), amount of contrast agent and reasons for the port systems´ removal.

### Statistical analysis

Descriptive data is provided as mean and standard deviation (SD) for normally distributed variables and as medians with ranges (minimum to maximum) for non-normalized variables. Categorical data is presented as counts and percentages. The Anderson-Darling test was used to assess normality, rejecting the hypothesis of normality when the *P*-value is less or equal to 0.05. The Kaplan-Meier method was used to analyze device service intervals free from infectious complications. Statistical analysis and the evaluation of the data were performed with a specialized computer algorithm (Microsoft Excel V1908 and RStudio 1.2.5033).

## Results

Technical success of the procedure was 97.1% (34/35 cases). In one case, the procedure was complicated due to post-thrombotic occlusion of the cephalic vein, however the catheter was successfully positioned centrally through existing collaterals. In another patient with Hodgkin lymphoma, unknown thrombosis of the subclavian vein hampered port implantation on the left; however, insertion of the port system on the right side was successful. Periprocedural complications such as venous spasm, bleeding or adverse reactions to medications or contrast agents were not observed.

Ports were implanted in the left forearm in 26 patients and in the right forearm in 9. For most procedures, the basilic vein was punctured for catheter introduction (28 procedures, 80%). In five instances, the cephalic vein was punctured (14.3%) and venous access in two patients was via the brachial vein (5.7%). In three patients, port implantation had to be performed twice. Two of these patients developed signs of port infection (on days 28 and 44 post implantation) and received repeated port implantation on the contralateral side after explantation, antibiotic therapy and normalization of infection parameters. One patient with recurrent catheter tip thrombosis underwent port system removal and implantation of a new port catheter system on the contralateral arm within one procedure.

Contrast medium was injected in 33/35 (94.3%) cases to verify correct catheter placement and connection, with a median volume of 5 ml and a range of 2 to 30 ml. In the other two cases, contrast medium was not given due to minor elevation of renal function parameters. Data regarding radiation exposure were available in 34/35 (97.1%) cases and yielded median procedural fluoroscopy times of 56.0 s (range: 9–264 s) and median dose area product values of 130.3 μGy·m^2^ (range: 7.8–1,284.3 μGy·m^2^) (Table 2).

**Table 2:** Procedural and outcome data (*n*=35)

Insertion side	*n*	%		
Left	26	74.3		
Right	9	25.7		
Location of insertion	*n*	%		
Basilic vein	28	80		
Cephalic vein	5	14.3		
Brachial vein	2	5.7		
Laboratory values on the day of the procedure	Mean/median	SD/min - max	Normal (%)	Abnormal (%)
CRP (mg/dl)	0.46	0.01–8.84	53.1	46.9
Leukocytes (10^9^/l)	8.4	5.4	57.1	42.9
Thrombocytes (10^3^/μl)	305	36–643	60.0	40.0
INR (%)	92	14.9	86.6	13.3
Radiation exposure data	Median	min - max		
Median fluoroscopy time (s)	56	9–264		
Median DAP (μGy·m^2^)	130.3	7.8–1,284.3		
Catheter duration time (days)				
Total	13,684			
Median	237			
Min	28			
Max	2,401			
Reasons for port removal (*n*=27)	n	%		
Completed therapy	18	66.7		
Patients’ request	2	7.4		
Port infection/wound healing disorder	6	22.2		
Thrombotic catheter occlusion	1	3.7		

*CRP* C-reactive protein, *DAP* dose area product, *INR* international normalized ratio, *max* maximum, *min* minimum, *SD* standard deviation

Within the observational period, 12 port systems were evaluated due to suspected thrombosis or system malfunction. This included clinical evaluation solely in two cases, ultrasound investigation in one case and fluoroscopic evaluation including contrast medium injection in nine cases.

**Fig. 2:**
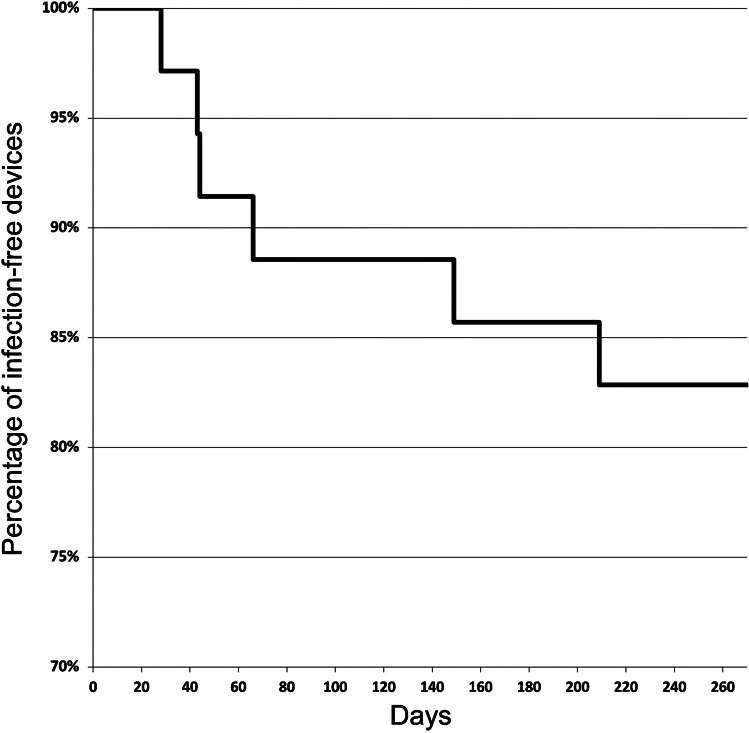
Kaplan-Meier curve of the overall infection-free port device survival

During the follow-up, 11 complications (31.4%) were documented, resulting in 0.8 complications per 1,000 catheter days, including 1 early complication (occurring within 30 days after implantation) and 10 late complications. The most frequent complications were infections and thrombosis (Table 3). The early complication was dehiscence of the wound related to catheter port insertion, necessitating port explantation. Concerning the late complications, in one case of port pocket infection and in four cases of catheter-associated bloodstream infection, the port system had to be removed. Three cases of catheter tip thromboses were managed with weight-adapted doses of low molecular weight heparin, whereas in one case the port system was removed. In one patient, ultrasound examination showed a catheter-related thrombotic occlusion of the basilic vein, which was successfully treated with heparin therapy. Material-related complications including catheter fracture, leakage or disconnection did not occur. In summary, the reported complications were classified as minor (grade B) in four cases (11.4%) and major (grade C) in seven cases (20%). Figure 2 illustrates overall infection-free catheter survival.

**Table 3:** Complications during follow-up

	*n*	/1,000 catheter days	(early^a^/late^b^)	Explantation necessary (*n*)
Catheter associated infection	4	0.3	(0/4)	4
Catheter tip thrombosis	4	0.3	(0/4)	1
Port pocket infection	1	0.07	(0/1)	1
Venous thrombosis	1	0.07	(0/1)	0
Wound healing disorder	1	0.07	(1/0)	1
Total	11	0.8	(1/10)	7
	*n*	%		
Early^a^	1	9.1		
Late^b^	10	90.9		

^a^Occurring within the first 30 days after the procedure

^b^Occurring more than 30 days after the procedure

Overall, the 35 port systems were implanted for a total of 13,684 days with a mean duration of 391 days (median: 237 days; range: 28–2,351 days). Within the follow-up period, 27 port systems (77.1%) were removed, including 17 radiologic and 10 surgical explantation procedures. In those cases of removal carried out in the Radiology Department, fluoroscopy was not used. In five patients, the port systems remained implanted and functioning until the end of the study period. Three patients died due to their underlying disease. The main reason for port system removal was completion of therapy (18/27; 66.7%). Removal-related complications did not occur.

## Discussion

With a technical success rate of 97.1%, our results are consistent with other larger studies proving a high technical success rate for the implantation of port systems in the upper arm or forearm [7]. Moreover, recent data confirm higher technical success rates in radiologic, imaging-guided and percutaneous implantation of port systems compared to open-surgical venous cutdown techniques [11]. Due to its minimally invasive nature, radiologic guided percutaneous insertion of TIVAP systems can be implemented in an outpatient setting. Further reasons for the increased transition of port catheter placements from surgical to radiologic techniques include lower complication rates and a favorable cost profile ([12–14]. The technique used exclusively in this study was venous puncture under ultrasound guidance. As an alternative, fluoroscopically-guided venous puncture following contrast agent injection via peripheral venous access has been reported [7]. We prefer the ultrasound-based approach due to the high level of control and safety during the puncture, while saving on radiation dose and avoiding administration of a contrast agent.

While one might expect complications during port implantation to occur more frequently in pediatric patients than in adults due to their smaller vessel sizes, we did not experience any procedure-related adverse events or complications impeding port catheter insertion, such as venous spasm or non-passable stenosis. However, complete failure of venous access occurred in one case with previously unknown thrombotic occlusion of the subclavian vein, necessitating the intraprocedural switch to the contralateral side. Furthermore, major procedure-related complications, such as severe vessel injury or arterial catheter placement, did not occur. The economical use of fluoroscopy is likely to reduce the risk of incorrect catheter placement to almost zero. The risk of pneumothorax, a potential complication of pectoral port implantation via the subclavian vein, can also be avoided when the venipuncture site is in the upper arm [15]. Thus, additional chest x-ray examinations after the procedure are avoided.

It may be hypothesized that pediatric patients are more prone to manual manipulation of the port chamber due to their age-related tendency to be more active and playful. Therefore, malposition of the systems´ components might be expected as a more common complication in this patient cohort. Indeed, the results of other studies suggest catheter dislodgement in pediatric patients occurs more frequently than in adults [16–18]. Severe complications like arrhythmia or vessel perforation might be the consequence. However, we did not experience cases of catheter malposition requiring revision in our study. Nor did we encounter port chamber malrotation, even though suturing for port chamber fixation within the subcutaneous pocket was not performed in our patients. However, it should be noted that we intentionally excluded patients of early childhood age, since we expected that infectious complications might occur more frequently in very young children due to their tendency to manipulate the easily reached site of port access in the forearm.

The impact of the implantation site and thus catheter length on the clinical outcome has revealed different, sometimes contradicting results. In this context, Fallon et al. [19] found higher rates of catheter migration and the need for operative revision when placed in a lateral inframammary position compared to a subclavicular or medial inframammary position. The authors hypothesized that the longer catheter length and the devices´ exposure to higher movement rates increase the risk for catheter displacement. In contrast, other large-scale retrospective studies revealed overall complication rates in peripherally inserted port systems in the forearm not exceeding those in chest ports [6, 7, 20].

A study published in 2002 found that longer catheter lengths and smaller diameters in peripherally inserted port systems are associated with higher rates of deep venous thrombosis and thrombotic catheter dysfunction [21]. Another study revealed port catheter-associated deep vein thrombosis to be a common finding on magnetic resonance venography in pediatric patients with cancer, with a thrombosis rate of 39.5% [22]. Nevertheless, it is worth mentioning that most of the reported deep vein thromboses remained clinically asymptomatic. In our study, investigation of port systems for thrombotic complications was not performed routinely, only in cases of malfunction or where there was clinical suspicion of thrombosis. Nor did we routinely perform pre-interventional ultrasound to identify unknown thrombosis, since subclinical thromboses are quite rare in children and adolescent patients. Instead, we evaluated history of thrombosis, as well as factors that could potentially increase the risk for thrombosis, during the informed consent conversation, and analyzed existing CT or MRI imaging for direct or indirect signs of thrombosis. With a total of 14.3%, the rate of thrombotic complications found in our cohort might be judged as high. However, it should be noted that, unlike other studies, catheter tip thromboses were also rated as thrombotic complications, even if the management did not include the systems´ removal. Another study from 2014 analyzing thrombotic complications in pediatric patients with pectoral port systems revealed symptomatic thrombosis in 20% of their patients [23]. Apart from the catheter access site, (with increased rates of thrombotic complications with the catheter in a subclavian position), further risk factors were not identified. Pharmacological prophylaxis did not appear to influence the thrombosis rate and there are no guidelines/standards recommending the regular use of any anticoagulation regimens to prevent catheter-related thrombosis [24]. A differential diagnosis of thrombus formation at the catheter tip is the presence of a fibrin sheath. The latter is indicated by difficulty aspirating blood from the catheter, whereas flushing/injection of fluid still remains possible [25].

Infection, the other relatively frequent complication associated with port systems, has been reported with varying incidences [20]. This is partially due to the differences in defining TIVAP-related bloodstream infection and in determining when to remove the port system [26]. Although some authors suggest lower infection rates in arm compared to chest ports, due to different extents of bacterial colonization between the two sites, most studies report similar rates of infectious complications [27]. Most infections found in our study occurred as a late complication. This underlines the importance of proper management of the port system, including sterile/aseptic handling and adequate saline flushing. Local infection of the port pocket or the tunnel area are rarely encountered [28]. The prophylactic administration of periprocedural antibiotics remains a matter of debate and due to conflicting evidence, there are no uniform recommendations [29, 30]. A recent study suggested the benefit of peri-interventional prophylactic antibiosis in reducing early and late infectious complications [31]. In our department, a single-shot prophylactic antibiotic is routinely administered to children with no history of antibiotic allergy and who are not already on antibiotics for other indications. This is in accordance with our hospital’s antibiotic regulations. Adverse effects related to antibiotic administration did not occur.

Although in most cases we used a normal-sized port system, we rarely experienced major complications such as wound dehiscence or skin erosions as reported by other authors [32]. However, it should be considered that only port systems in an upper arm position were evaluated in the latter study. Varying amounts of subcutaneous tissue between the upper arm and forearm might result in different complication rates by impacting the systems´ accessibility and wound healing.

In our moderate-sized study, seven (20%) complications necessitating port system removal occurred. These results are comparable to those of the few other studies that dealt with port-associated complications in children, although it is noteworthy that most articles in the current literature specifically focus on a single complication when assessing port systems in children [17]. Removal-associated complications like catheter dislodgement and irremovable catheters have been reported in open surgical and percutaneous implanted port systems in children, sometimes necessitating venotomy or a further intervention [33]. However, these particular complications did not occur in our cohort.

Data regarding radiation exposure related to implantation of central venous port systems are extremely sparse. With a median DAP of 130.3 μGy·m^2^ and median fluoroscopy times of 56 s, the radiation doses found in our study are lower than those that have been reported in a study from 2006, analyzing 303 pectoral port implantation procedures (median DAP: 373 μGy·m^2^; median FT: 210 s) [34]. In contrast, our procedure-related radiation doses exceed those of a study by Jonczyk et al. [35] from 2018, in which median DAP values of 57.3 μGy·m^2^ and median FT values of 24 s were reported in port implantation in a pectoral position performed by senior radiologists. In that study, authors found decreased radiation doses for jugular venous access compared to subclavian access routes and in right-sided jugular accesses compared to left. Higher FT and DAP are likely when visualizing the significantly longer catheter route in peripherally inserted arm ports. However, in view of the increased sensitivity of children to radiation exposure, the lowest possible intraprocedural radiation doses should be aimed for. The comparatively wide range of procedure-related radiation doses documented in our study might be due to the lack of standards in image documentation. Instead, image acquisition was left to the discretion of the performing interventional radiologist and thus, in some cases, the result of port implantation was documented by peripheral and central digital subtraction angiography series, whereas in others only fluoroscopic single images were stored. Further reductions in radiation dose might also be achieved by consistent utilization of the “last-image-hold” function. Not surprisingly, higher radiation doses were documented in technically difficult procedures, for example due to post-thrombotic venous complications, as well as in patients with increased body mass index. Unfortunately, to date, dose reference levels for this procedure do not exist.

The choice of implantation site is influenced by the potential for patient discomfort. A previous study documented less discomfort with venous access ports implanted in the forearm compared to pectoral ports [36]. Furthermore, patients with venous access ports in the chest have been reported to experience discomfort more frequently when using seat belts or wearing a bra [37]. Location of port devices in a pectoral position might lead to relatively more discomfort in younger female patients. In this patient group, easy access via the forearm, comparable to standard peripheral venous access, might be an attractive alternative. Cosmetic factors, with avoidance of scar formation in the cleavage, might additionally favor implantation of port systems in a forearm position, especially in young patients. We subjectively experienced high levels of patient satisfaction in terms of daily activity and general comfort associated with implantation in the forearm position. However, we did not conduct an analysis of pediatric patients´ opinions. Structured questionnaires would contribute to our further understanding of the patient’s perspective and should be an aim of further studies.

The main limitations of this study are its retrospective, non-randomized design and the limited number of included patients. To develop general recommendations, a larger patient population would be ideal. Nevertheless, the present study is the largest to date on adolescents receiving ultrasound-guided TIVAPs in the forearm. Another limitation is the lack of children below 9 years of age (older than in other studies). The technique used in this study, particularly venous access, presumably becomes more difficult the smaller the patient. However, considering the small-sized port systems available on the market today, there should be no definitive cutoff age for this implantation site and studies in younger children are warranted.

## Conclusion

Peripheral and percutaneous implantation of venous access ports in the forearm in children and young adults has a high technical success rate and complication rates are comparable to those in adult cohorts.
